# 16S rRNA metagenomics data on the bacterial communities in integrated poultry-fish farm ponds

**DOI:** 10.1016/j.dib.2022.108269

**Published:** 2022-05-15

**Authors:** Sunday B. Akinde, Folasade M. Adeyemi, Omotayo O. Oyedara, Temitope F. Ajani, Waidi F. Sule, Janet O. Olaitan, Bamidele A. Iwalokun, Olabisi O. Ojo

**Affiliations:** a Department of Microbiology, Faculty of Basic and Applied Sciences, Osun State University, Osogbo, Nigeria; b Departamento de Microbiología e Inmunología, Facultad de Ciencias Biológicas, Universidad Autónoma de Nuevo León, San Nicolás de los Garza, Nuevo León, 66455, Mexico; c Molecular Biology and Biotechnology Department, Nigerian Institute of Medical Research, Lagos, Nigeria; d Central Research Laboratory, Nigerian Institute of Medical Research, Lagos, Nigeria; e Department of Natural Sciences, Albany State University, Albany, Georgia, USA

**Keywords:** Integrated poultry-fish farm, Sequencing, 16S rRNA gene, Bacterial diversity, Nigeria

## Abstract

In an integrated poultry-fish (IPF) farming system, fish and bird are reared simultaneously. It is a common practice in Sub-Saharan Africa countries like Nigeria, Cameroon, Madagascar, and Benin, offering economic benefits to farmers and minimizing farm running costs. It seems like another way for farmers to manage poultry waste as it is a common practice in IPF farm settings to feed reared fishes with wastes emanating from the poultry. This work provides dataset on the bacterial taxonomic profile and abundance in IPF farm pond water samples using the 16S rRNA sequencing approach. Using ZymoBIOMICS®-96 MagBead DNA Kit, total DNA was extracted from pond water samples collected from IPF farm located at Ila-Orangun, Osun State, Southwest Nigeria (Long: 8° 1′ N; Lat: 4° 54′ E) during two sampling visits. The V3-V4 region of the rRNA gene was amplified and sequenced on the Miseq Illumina sequencing platform. Raw reads obtained after demultiplexing were analyzed using DADA2 pipeline to obtain distinctive or unique amplicon sequence variants which were grouped into Operational Taxonomic Units (OTUs) based on similarities. Taxonomy assignment was performed using UCLUST and Bayesian classifier from QIIME v.1.9.1 with the Zymo Research Database as reference. The phyla Proteobacteria (26.7%), Actinobacteria (26.0%), Firmicutes (13.1%), and Cyanobacteria (10.1%) dominated the 35 phyla obtained from the OTUs. Interestingly, the abundance of bacterial pathogens commonly associated with human infections was low. The sequence and sample data have been deposited in NCBI database under Sequence Read Archive (SRA) with Bioproject identification number PRJNA760919 (Accession number: SRX12020336 – SRX12020346). The dataset obtained can bridge the gap of limited information on the impact of IPF farming on pond bacterial diversity, a critical factor for considerations as regards food safety, fish, and public health.

## Specifications Table

**Table:** 

Subject	Environmental Genomics and Metagenomics
Specific subject area	Metagenomics
Type of data	16S rRNA read sequences, table, graph
How the data were acquired	The V3-V4 region of the rRNA gene was amplified and sequenced on the Miseq Illumina sequencing platform. Bioinformatic tools used for the sequence analysis include DADA2 pipeline, QIIME v.1.9.1 and LEfSe algorithm.
Data format	Raw and Analyzed data
Description of data collection	Water samples were collected from two integrated ponds and one unintegrated pond constructed on an IPF farm during two sampling visits. DNA was extracted from the samples using ZymoBIOMICS®-96 MagBead DNA Kit and subjected to Miseq Illumina sequencing platform using a 500 cycles v3 sequencing kit (2 × 250 bp paired-end reads). Distinctive or unique amplicon sequence variants obtained using the DADA2 pipeline was grouped into OTUs and taxonomy assignment was performed using QIIME v.1.9.1. Taxonomy with significant abundance within different groups was detected by LEfSe algorithm using default settings.
Data source location	Institution: Integrated poultry-fish farm City/Town/Region: Ila-Orangun/ South-west Country: Nigeria Latitude and longitude: Long: 8° 1′ N; Lat: 4° 54′ E
Data accessibility	The sequence and sample data have been deposited in NCBI database under Sequence Read Archive (SRA) with Bioproject identification number PRJNA760919 (Accession number: SRX12020336 – SRX12020346). The raw data is deposited in the mendeley data repository. URL: https://data.mendeley.com/drafts/xvt7v7wp56 DOI: http://dx.doi.org/10.17632/xvt7v7wp56.2

## Value of the Data

• There is a paucity of data on the bacterial community associated with pond water used for IPF farming practices. The datasets obtained provide information on the bacterial diversity and abundance in IPF farm ponds. The data could be useful in monitoring the potential public health impact of IPF farming system.
• Since IPF farming system is a common practice in Nigeria due to its economic benefits, the data can provide baseline information that could guide farmers and policy makers in the management of IPF activities and formulation of policies that can prevent any negative outcome of the practice, such as outbreaks.
• The taxonomic profile dataset could be useful for researchers who are interested in understanding the cascade effects (horizontal gene transfer, microbiome alteration, among others) of interaction between the poultry and fish pond microbiomes in an IPF farm settings. The comparison of this dataset with taxonomic profile obtained from farms that do not engage in IPF farming system could also guide in advocating best agricultural practices devoid of public health challenges.

## 1 Data Description

**Fig. 1:**
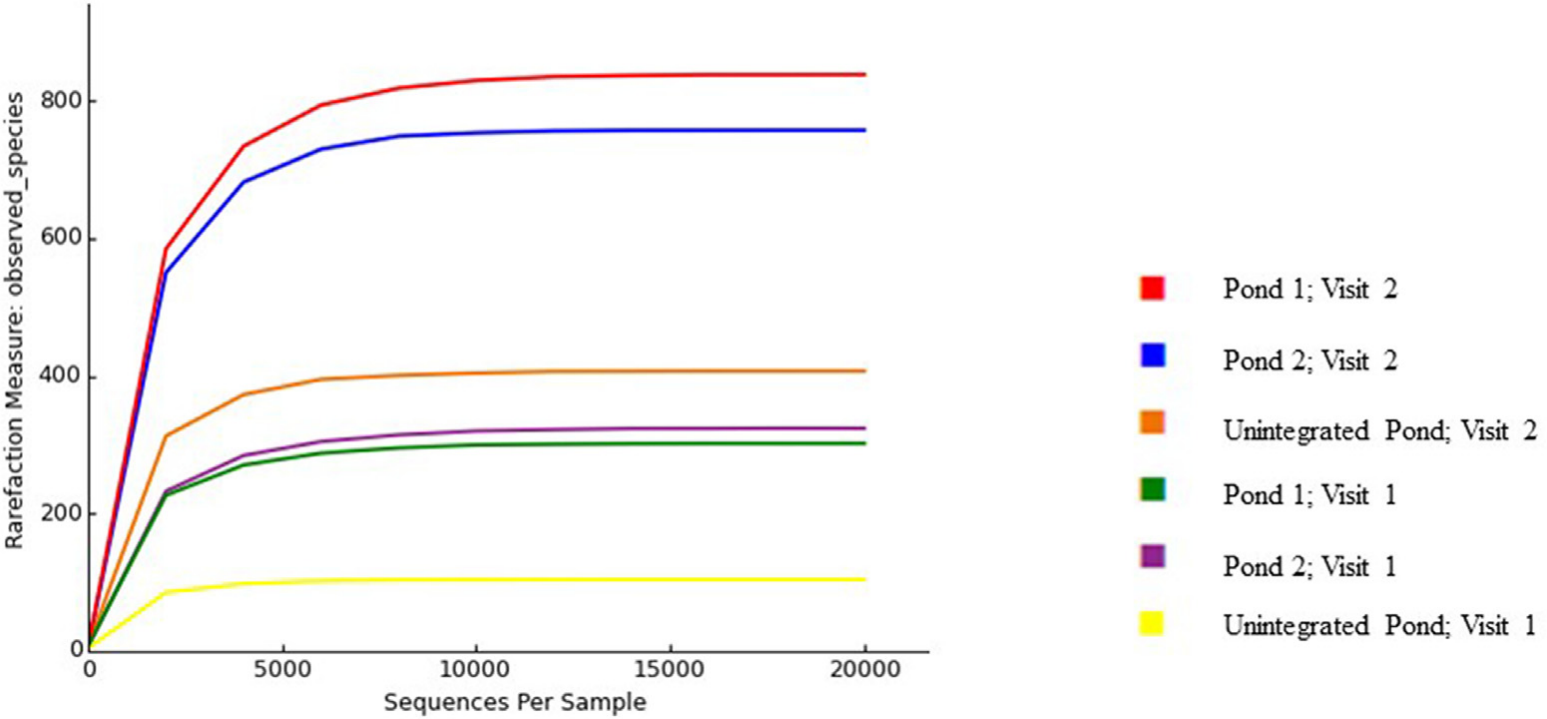
The rarefaction curve shows the sequence and richness coverage of OTUs present in each pond water sample.

**Fig. 2:**
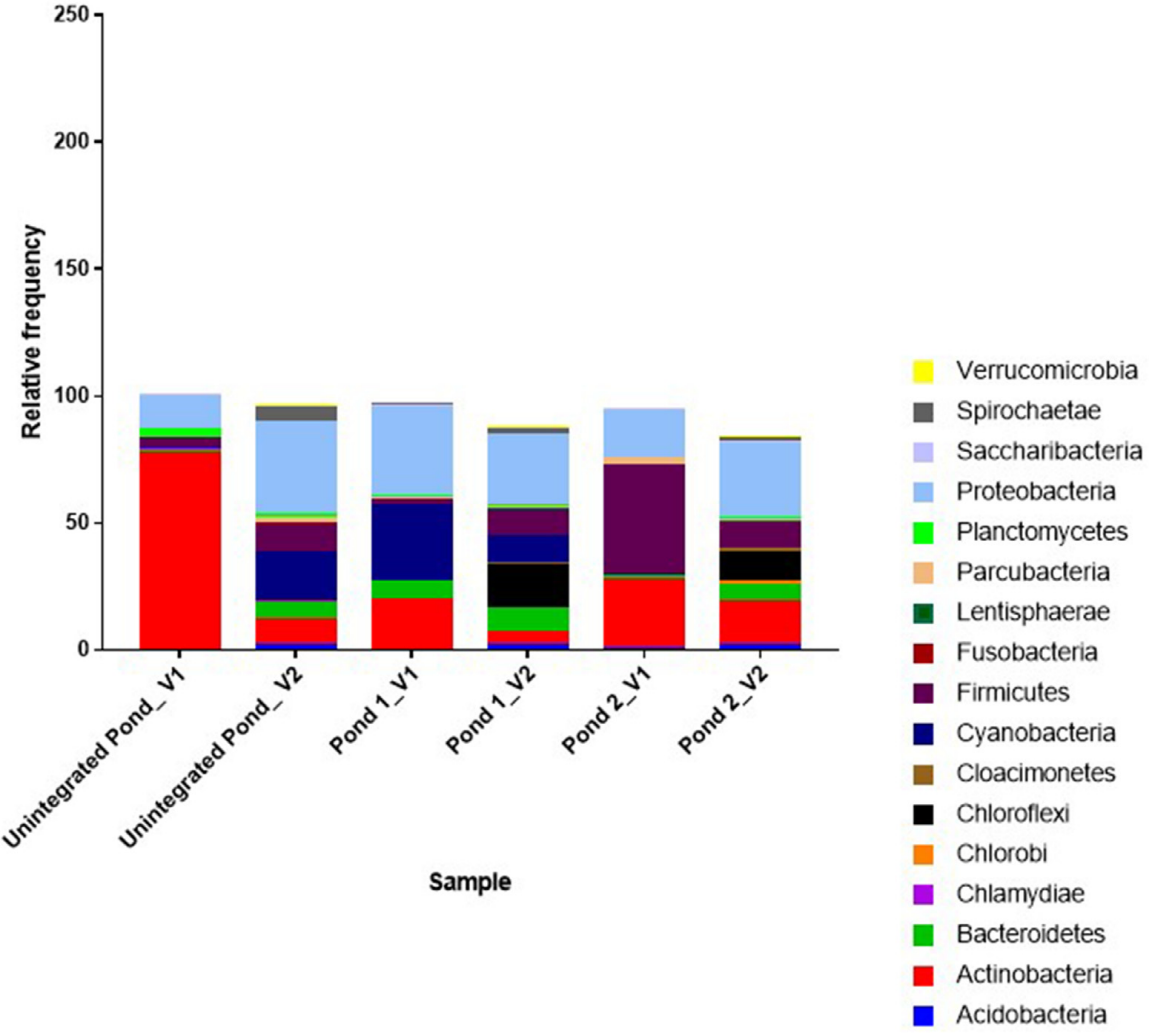
The relative frequency of the most abundant phyla identified in the pond water samples collected from the integrated poultry-fish farm. V1 and V2 indicate visit 1 and 2, respectively.

The dataset in this study provided information on the bacterial diversity of pond water used for the IPF farming. Water samples were collected from two integrated ponds and one unintegrated pond constructed on an IPF farm during two sampling visits. A total of 638,492 raw read sequences were processed from the six pond water samples to obtain a total of 2,732 unique OTUs (Table 1). The rarefaction curve, which represents species richness, approached a plateau for each sample, indicating that the sequencing captured most of the abundant bacterial species, and further sequencing would not lead to the detection of new haplotypes or bacterial species (Fig. 1). During the two visits, the highest number of unique OTUs clusters was observed in the water from integrated pond 1 (302 and 838), followed by pond 2 (324 and 757), and then the unintegrated pond water (104 and 407). The OTUs in the samples were grouped into 35 phyla (Fig. 2), and the most frequently occurring phyla overall were Proteobacteria (26.7%), Actinobacteria (26.0%), Firmicutes (13.1%), and Cyanobacteria (10.1%). During the first visit (in April), the most abundant phyla in the unintegrated pond were Actinobacteria (77.6 %), while the phylum Proteobacteria were found dominant (36.4 %) during the second visit (in August). Furthermore, the phyla Actinobacteria, Proteobacteria, Planctomycetes, Firmicutes, and Chlorobi dominated in the unintegrated fish pond in April. But dominance by new phyla such as Cyanobacteria, Bacteroidetes, Spirochaete in the same unintegrated fish pond in August were observed. Apart from the emergence of new phyla in the unintegrated fish pond in August, the decline in the abundance of Actinobacteria and Planctomycetes phyla was also observed. These findings indicate changes in diversity and abundance as well as dynamics of bacterial populations despite that the aquaculture fish pond was not exposed to poultry wastes. The phylum Firmicutes (42.2 %) were more abundant in pond 2 during the first visit, while phylum Proteobacteria (29.2 %) dominated during the second visit to the farm. In addition, phyla Acidobacteria Verrucomicrobia, spirochaetae, and Chloroflexi were observed in pond 1 and 2 during the second visit.

**Table 1:** The sequences obtained from pond water samples collected at the integrated poultry-fish farm representing the OTUs present in the samples.

S/No	Pair-end sample sequence	Raw reads	Number of OTUs	SRA Accession number
First Visitation to the sampling site				

1a	Pond 1 (Read 1)	105072	302	SRX12020336
b	Pond 1 (Read 2)			SRX12020337
2a	Pond 2 (Read 1)	125532	324	SRX12020342
b	Pond 2 (Read 2)			SRX12020343
3a	Unintegrated pond (Read 1)	92724	104	SRX12020346
b	Unintegrated pond (Read 2)			SRX12020347

Second visitation to the sampling site				

4a	Pond 1 (Read 1)	112170	838	SRX12020340
b	Pond 1 (Read 2)			SRX12020341
5a	Pond 2 (Read 1)	101314	757	SRX12020344
b	Pond 2 (Read 2)			SRX12020345
6a	Unintegrated pond (Read 1)	101680	407	SRX12020338
b	Unintegrated pond (Read 2)			SRX12020339

	Total	638,492	2,732

**Fig. 3:**
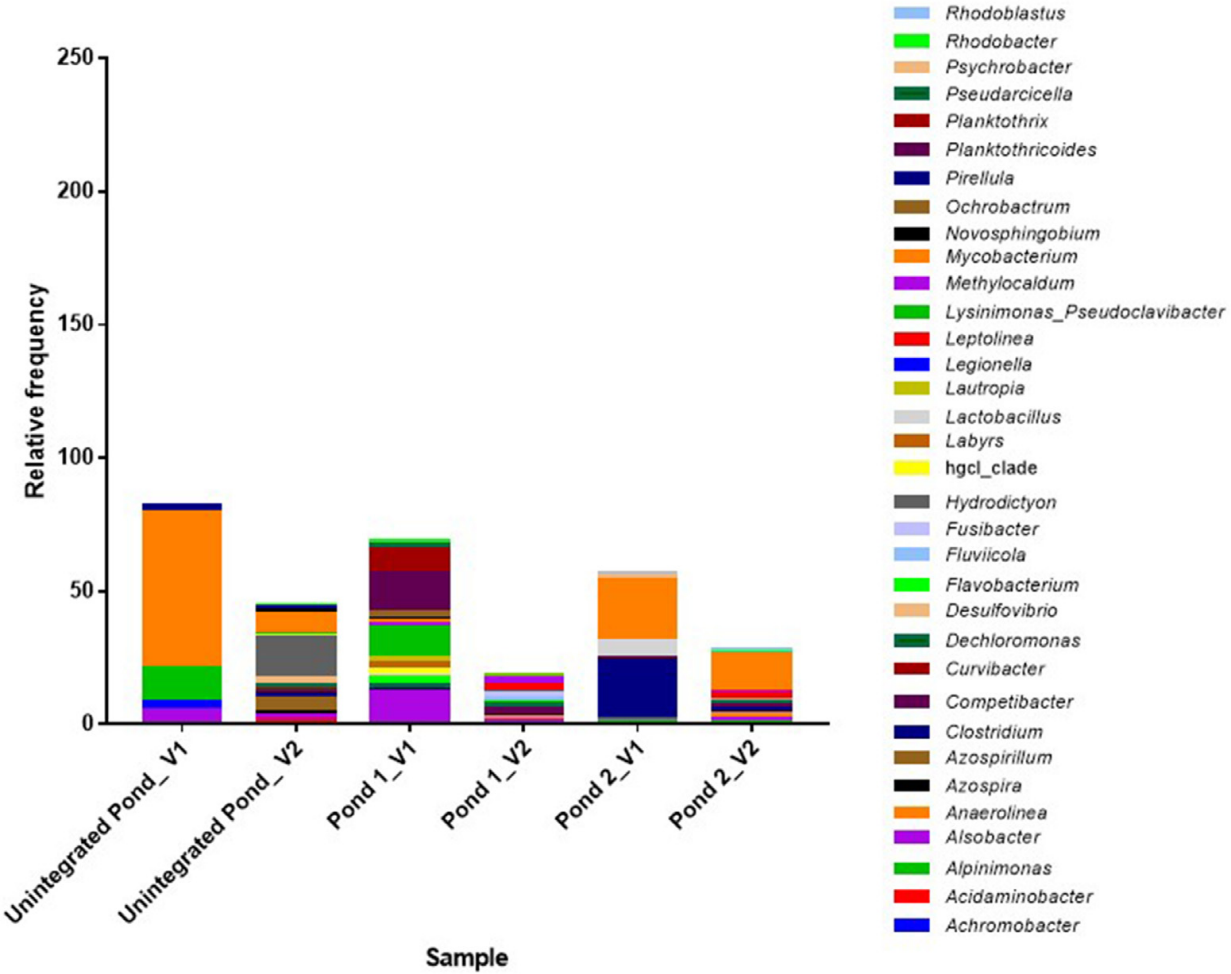
The relative frequency of the most abundant genera identified in the pond water samples collected from the integrated poultry-fish farm. V1 and V2 indicate visit 1 and 2, respectively.

**Fig. 4:**
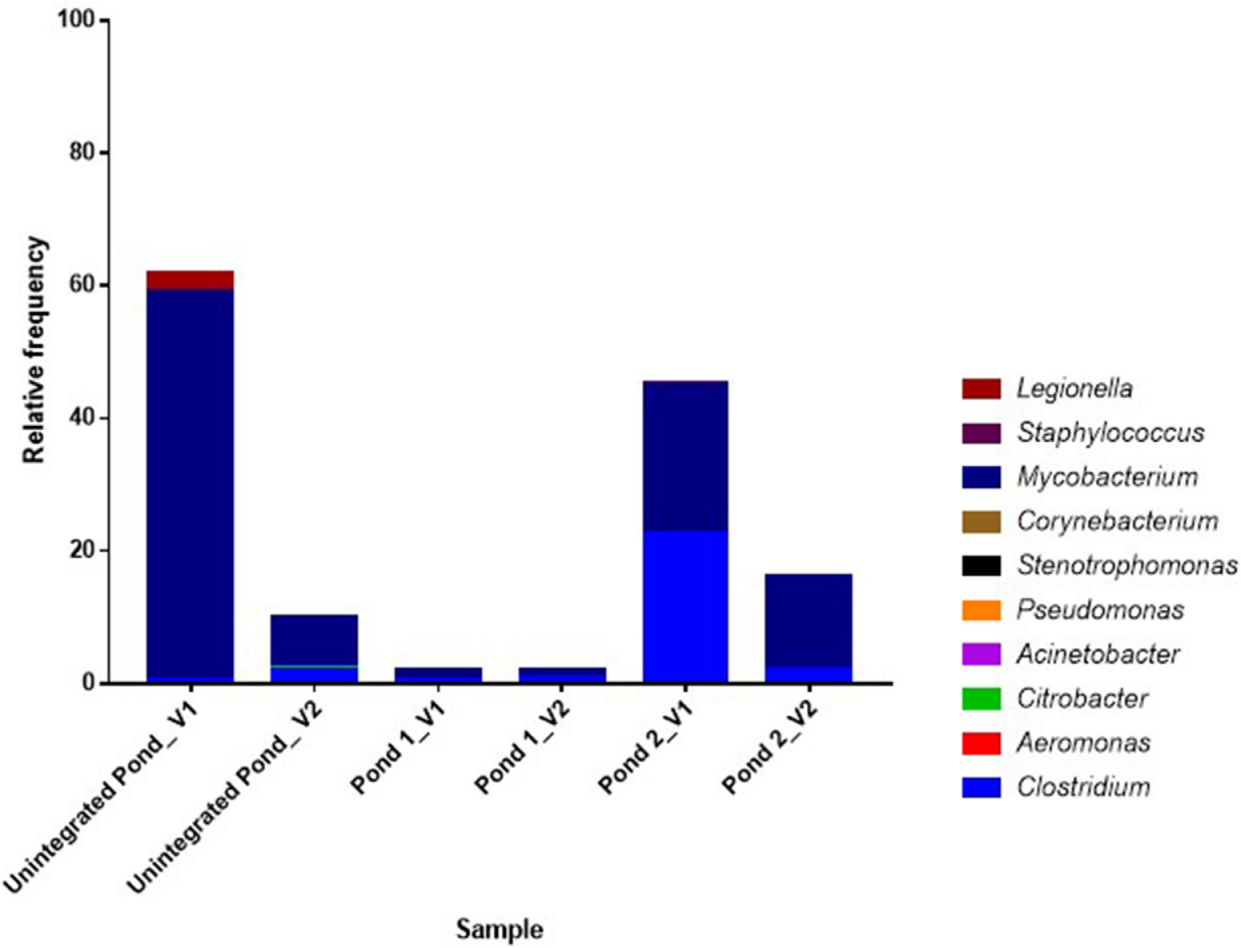
The abundance of bacterial genera that have been reported as pathogens identified in the pond water samples. V1 and V2 indicate visit 1 and 2, respectively.

The most abundant bacterial genera identified in OTUs are presented in Fig. 3. Except for the genera *Mycobacterium* and *Clostridium*, all members of the genera that have been implicated in reports as potential pathogen were detected at low abundance (Fig. 4.). The raw read sequences were deposited in the NCBI database under the sequence read archive (SRA) with Bioproject identification number PRJNA760919 (https://www.ncbi.nlm.nih.gov/sra?linkname=bioproject_sra_all&from_uid=760919). The data file including the alpha diversity plot, which is the measurement of unique sequences or OTUs richness and evenness of each pond water based on four estimators (shannon, chao1, observed species, and phylogenetic distance indices), rarefaction plots for the different alpha diversity estimators, and absolute abundance of bacterial DNA in the study ponds was deposited in the mendeley data repository (https://data.mendeley.com/drafts/xvt7v7wp56).

## 2 Experimental Design, Materials and Methods

### 2.1 Study area and Sample collection

Water samples were collected from two integrated ponds and one unintegrated pond constructed on an IPF farm located at Ila-Orangun, Osun State, South-west Nigeria (Long: 8° 1′ N; Lat: 4° 54′ E) during two sampling visits in April and August 2019. The farm rears catfish (*Clarias* spp) and exotic breed of layer chickens, with populations of 25,000 and 22,600, respectively. The integrated pond refers to a pond that directly/indirectly received poultry wastes to feed fishes, while the unintegrated pond did not have contact with poultry wastes. Pond water samples (1L) were collected into a 1L sterilized plastic bottle using a sterilized stainless steel water sampler. The samples were transported on ice inside a sample box to Osun State University, Microbiology laboratory, Osogbo. Using a vacuum pump membrane filtration unit (Microlab Scientific Co. Ltd Zhejiang Province, China), 50 mL each of the pond water samples was filtered using 0.22 µm filter paper (MF- Millipore^TM^ Membrane, Merck Millipore Ltd, Tullagreen, Carrigtwohill, Co.  Cork, Ireland). The filters were preserved with DNA/RNA shield reagent (Zymo Research, Irvine, CA) and stored at –20 °C for subsequent analyses.

### 2.2 DNA extraction and targeted library sequencing

Metagenomic DNA was extracted using ZymoBIOMICS®-96 MagBead DNA Kit (Zymo Research, Irvine, CA). Quality assessment of the extracted DNA was performed by fluorometry using Qubit 4.0. Bacterial 16S ribosomal RNA gene targeted sequencing was performed using the Quick-16S™ NGS Library Prep Kit (Zymo Research, Irvine, CA), with bacterial 16S primers which amplified the V3-V4 region of the 16S rRNA gene. The barcoded rRNA amplicon libraries were then subjected to sequencing on the Miseq Illumina sequencing platform (Illumina, USA) using a 500 cycles v3 sequencing kit (2 × 250 bp paired-end reads). The raw reads of different samples were subsequently generated after demultiplexing.

### 2.3 Bioinformatics analysis

Using the DADA2 pipeline [1], distinctive or unique amplicon sequence variants were extrapolated from raw reads and grouped into Operational Taxonomic Units (OTUs) based on similarities after chimeric sequences were removed. Taxonomy assignment was performed using UCLUST and Bayesian classifier from QIIME v.1.9.1 [2] with the Zymo Research Database as reference. Taxonomy with significant abundance within different groups was detected by LEfSe [3] using default settings.

## CRediT authorship contribution statement

**Sunday B. Akinde:** Conceptualization, Methodology, Formal analysis, Investigation, Resources, Supervision. **Folasade M. Adeyemi:** Writing – original draft. **Omotayo O. Oyedara:** Methodology, Formal analysis, Investigation, Writing – original draft, Writing – review & editing. **Temitope F. Ajani:** Methodology, Formal analysis, Investigation. **Waidi F. Sule:** Writing – original draft. **Janet O. Olaitan:** Writing – review & editing. **Bamidele A. Iwalokun:** Writing – review & editing. **Olabisi O. Ojo:** Conceptualization, Methodology, Formal analysis, Investigation, Writing – review & editing, Resources, Supervision.

## Declaration of Competing Interest

The authors declare that they have no known competing financial interests or personal relationships that could have appeared to influence the work reported in this paper.

## Data Availability

16S rRNA Metagenomics Data on the Bacterial Communities in Integrated Poultry-Fish Farm Ponds (Original data) (Mendeley Data).
